# Reliability and predictive validity of a hepatitis-related symptom inventory in HIV-infected individuals referred for Hepatitis C treatment

**DOI:** 10.1186/1742-6405-8-29

**Published:** 2011-08-10

**Authors:** Edward R Cachay, David L Wyles, Miguel Goicoechea, Francesca J Torriani, Craig Ballard, Bradford Colwell, Robert G Gish, William C Mathews

**Affiliations:** 1 Department of Medicine, University of California at San Diego. 200 West Arbor Drive, San Diego, California 92103. USA; 2 Department of Pharmacy, University of California at San Diego. 200 West Arbor Drive, San Diego, California 92103. USA; 3 Department of Gastroenterology and Hepatology, University of California at San Diego. 200 West Arbor Drive, San Diego, California 92103. USA

## Abstract

**Background:**

We aimed to determine the reliability and validity of a hepatitis symptom inventory and to identify predictors of hepatitis C (HCV) treatment initiation in a cohort of HIV-infected patients.

**Methods:**

Prospective clinic based study that enrolled patients referred for HCV therapy consideration. A hepatitis symptom inventory and the Center for Epidemiologic Studies Depression Scale (CES-D) were administered to HIV/HCV individuals. The symptom inventory was factor analyzed and subscale reliability estimated with Cronbach's alpha. Predictive validity was evaluated using generalized estimating equations (GEE). Predictors of HCV treatment were identified using logistic regression.

**Results:**

Between April 2008 to July 2010, 126 HIV/HCV co-infected patients were enrolled in the study. Factor analysis using data from 126 patients yielded a three-factor structure explaining 60% of the variance for the inventory. Factor 1 (neuropsychiatric symptoms) had 14 items, factor 2 (somatic symptoms) had eleven items, and factor 3 (sleep symptoms) had two items, explaining 28%, 22% and 11% of the variance, respectively. The three factor subscales demonstrated high intrinsic consistency reliability. GEE modeling of the 32 patients who initiated HCV therapy showed that patients developed worsening neuropsychiatric and somatic symptoms following HCV therapy with stable sleep symptoms. Bivariate analyses identified the following as predictors of HCV therapy initiation: lower HIV log_10 _RNA, lower scores for neuropsychiatric, somatic and sleep symptoms, lower CES-D scores and white ethnicity. In stepwise multiple logistic regression analysis, low neuropsychiatric symptom score was the strongest independent predictor of HCV therapy initiation and HIV log_10 _RNA was inversely associated with a decision to initiate HCV treatment.

**Conclusions:**

A 41-item hepatitis-related symptom inventory was found to have a clinically meaningful 3-factor structure with excellent internal consistency reliability and predictive validity. In adjusted analysis, low neuropsychiatric symptom scores and controlled HIV infection were independent predictors of HCV treatment initiation. The usefulness of the HCV symptom inventory in monitoring HCV treatment should be evaluated prospectively.

## Background

Hepatitis C (HCV) co-infection in HIV-infected patients has a more rapid progression to liver fibrosis, cirrhosis, and ultimately death despite well controlled HIV infection [1-3]). Thus, HCV has become a priority in the care of HIV individuals co-infected with HCV in USA and Europe since 2005 [4,5]. However, the proportion of co-infected HCV/HIV individuals who initiate HCV therapy remains less than 25% in the USA after more than five years of available dual therapy with pegylated interferon and ribavirin [6,7]. Multiple medical and psychosocial barriers that HIV patients face at any given time in their care accounts for this low proportion of HCV treatment initiation [8-10]. Furthermore, the frequency and severity of depression are more prevalent among HCV/HIV patients [11,12]. Current standard protocols for the assessment of HCV treatment eligibility incorporate routine screening of depression [13], However, HCV/HIV patients often have multiple non-specific symptoms referred as 'somatic symptoms' that may or not be related to hidden depression [12]. Of note, up to 47% of HCV/HIV patients discontinue therapy due to worsening of underlying symptoms aggravated by HCV therapy [14-16]. Currently there is lack of standardization for assessment of these symptoms among HCV/HIV patients being considered for HCV treatment initiation.

In April 2008, a multidisciplinary hepatitis-HIV co-infection program was established at the University California, San Diego (UCSD) as part of a comprehensive HIV primary care system (Owen Clinic). We recognized that HCV treatment side effects may influence treatment initiation decisions and also affect treatment adherence and completion [17]. We therefore set out an exploratory analysis to evaluate a HCV symptom inventory in a population of HIV-coinfected patients referred for HCV treatment. The aims of this study were: 1) to determine the reliability and validity of a HCV symptom inventory and 2) to identify predictors of HCV treatment initiation in a cohort of HIV patients referred for HCV treatment.

## Methods

### Study design

This prospective study was conducted on patients referred to the UCSD Owen Hepatitis Co-infection clinic for the assessment of HCV treatment eligibility. The psychometric properties of the HCV symptom inventory were studied using data from this longitudinal cohort. The study was approved by the UCSD Human Research Protection Program (project# 071931). All patients upon Owen Clinic enrollment provided written informed consent for the collection of data relevant to their clinical care and subsequent analysis. This study was conducted according to the principles expressed in the Declaration of Helsinki.

### Inclusion criteria and study enrollment

Patients diagnosed with HCV/HIV co-infection either by HCV ELISA antibody and/or HCV polymerase chain reaction were referred to the UCSD Owen Hepatitis Co-infection Clinic for staging of HCV infection and assessment of treatment eligibility. Participants enrolled in the present study were required to: (1) read and understand English; (2) fulfill minimal clinical criteria for HCV treatment eligibility according to our clinic protocol (see below); (3) complete HCV clinical staging process; and (4) be willing to complete study survey instruments.

### Clinical assessment of Hepatitis C treatment eligibility

Our minimal requirements for HCV treatment eligibility were: (1) undetectable HIV viral loads and CD4 cell counts above 200/cm^3 ^if on antiretroviral therapy; (2) CD4 cell count above 500/cm^3 ^irrespective of HIV viral load value if naïve to antiretroviral therapy; (3) absence of liver cirrhosis; (4) stable medical comorbidities; (5) favorable recommendation from the team's psychiatrist who performed independent evaluations of every patient regardless of prior psychiatric history; (6) registration in the San Diego needle exchange program in the case of ongoing parenteral illicit substance use with documentation of controlled HIV infection as in (1) and (2), plus documentation of no missed clinic appointments during HCV staging process; and (7) alcohol sobriety for at least 6 months prior to HCV treatment initiation.

Clinical HCV treatment initiation decisions were made following multidisciplinary review of medical, psychiatric, social and substance abuse assessments. Psychiatric care included pre-emptive use of antidepressants when indicated. Treatment initiation decisions were made, however, without clinician knowledge of HCV symptom inventory scores.

### Study procedures and data collection

After recording of vital signs, a patient was placed in the exam room. While the patient waited to be seen by one of our team clinicians, our substance counselor met with the patient, explained and administered a computer assisted survey. If the patient had additional questions, their clinicians answered these queries; prior to the patient's final completion of the computer assisted inventory. The survey was administered online using Remark Web Survey version 5.0 (Gravic Inc., Malvern, Pennsylvania) using the exam room computer. The first page of the survey contained the Center for Epidemiologic Studies Depression Scale (CES-D) questions. The second page contains the hepatitis symptom inventory questions. Each symptom item includes a 6-category, Likert scaled symptom severity response, ranging from 0 (symptom absent) to 5 (symptom very severe). No other battery of questions was administered. Surveys were self-administered by patients and took, on average, five minutes to complete. It was the responsibility of the evaluating clinician to verify that willing patients completed the symptom inventory. Data was stored on a secure intranet server of the study clinic.

Electronic medical records from each study participant were reviewed to abstract information regarding demographic characteristics, substance use, psychiatry history, and relevant clinical data.

### Instruments and frequency of measurement

The 41-HCV symptom inventory was chosen because it was already in use as monitoring tool of side effects of HCV therapy in an unselected HCV treatment population [18]. The HCV symptom inventory is a modified version of the "Neurotoxicity Rating Scale" [19]. This scale includes items scored from 0 ("not present") to 4 ("extremely severe") measuring the severity of various psychiatric, cognitive, neurovegetative and somatic symptoms known to be induced by interferon therapy [19]. Scores range from 0 to 148; the mean [± SD] score in patients in a prior study who discontinued interferon alfa therapy due to severe depression or neurotoxicity was 42.6 ± 26.2 [20].

The CES-D is a 20-item, self-report, depression inventory with each item scored on a scale of 1-4 (higher scores indicate more depressive symptoms). The CES-D has been validated among HCV infected people and was shown to have a four-factor structure: negative effect, positive effect, depressed effect, and somatic, with the last factor being composed of 3 items. Total score can range from 0 to 60; a score of 16 or greater generally is considered the cutoff score associated with depressive symptoms [21].

Both the Center for Epidemiologic Studies Depression Scale (CES-D) and HCV symptom inventory were administered during the first clinic visit (baseline) and at subsequent study clinic visits of the HCV clinical staging process. Patients who subsequently initiated HCV therapy were seen at least monthly according to our clinic protocol with completion of surveys at every clinic visit.

### Statistical analysis

Continuous variables are expressed as means ± SD. Differences between groups were compared using the Mann-Whitney U test and Fisher's exact tests for continuous and categorical values, respectively. Principal component factor analysis with varimax rotation was performed to determine the factor structure of the HCV symptom inventory and to develop subscales of suitable internal consistency and reliability. Only factors with eigenvalues greater than 1.5 were retained for analysis. An item required factor loadings greater than 0.50 in order to be included in a subscale. Subscale reliability was estimated with Cronbach's alpha. Convergent validity of the HCV symptom inventory was assessed by examining the joint distribution and correlation between the HCV symptom inventory (total and subscale scores) and a previously validated depression screening tool (CES-D). Differences between dependent correlations involving CES-D and each of the symptom subscales were estimated using bootstrap resampling with 1000 replications (Stata *bootcor *procedure). Predictive validity of HCV symptom inventory was assessed in two ways: (1) in the prediction of the clinical decision to initiate HCV treatment based on pre-treatment assessment; and (2) in longitudinal changes in HCV symptom inventory scores while on therapy using generalized estimating equations (GEE). The null hypotheses for both predictive validation components were, respectively, that: (1) symptom scores were unrelated to treatment decisions after taking into account established indications for treatment initiation; and (2) HCV symptom inventory scores do not change on HCV treatment. To identify baseline predictors of HCV treatment initiation we used analysis of variance and Fisher's test for continuous and categorical variables, respectively. Significant (p > 0.10) bivariate predictors were entered into stepwise multiple logistic regression analyses. Statistical significance for all tests was defined as a two-tailed p < 0.05. Analyses were performed using Stata version 11.0 (Stata Corp., College Station, Texas, USA).

## Results

Between 1 April 2008 and 31 July 2010, 193 HIV-infected individuals naïve for HCV therapy were referred to our clinic for assessment of HCV treatment eligibility. Sixty-seven patients were not enrolled in the study for the following reasons: (1)cirrhosis and evidence of portal hypertension (n = 17); (2) declined staging procedures and preferred to wait for new HCV therapies (n = 15); (3) omission of survey completion during early months on study implementation (n = 13); (4) declined any medical treatment or evaluation (n = 9); (5) HCV antibody positive and HCV RNA negative and did not need HCV therapy (n = 7); and (6) transferring care to a different city and clinical staging could not be completed (n = 6). The final study sample was 126 HCV/HIV co-infected patients that completed the HCV symptom inventory and clinical staging process for HCV treatment eligibility. Median age was 49. By HIV risk factor, 41% were intravenous drug users, 37% men who have sex with men, and 14% acquired HIV through heterosexual transmission. By HCV risk factor, 75% were intravenous drug users, 23% men who had sex with men and 2% hemophilia patients. Most patients (59%) had prior diagnosis of major depression or bipolar disorder, of whom 31% were taking psychotropic medications at time of medical of assessment. Active use of illicit drugs was reported by 15% of patients, most of them reporting intravenous drugs (15 of 19 patients).

### HCV symptom inventory factor structure and convergent validity

**Figure 1:**
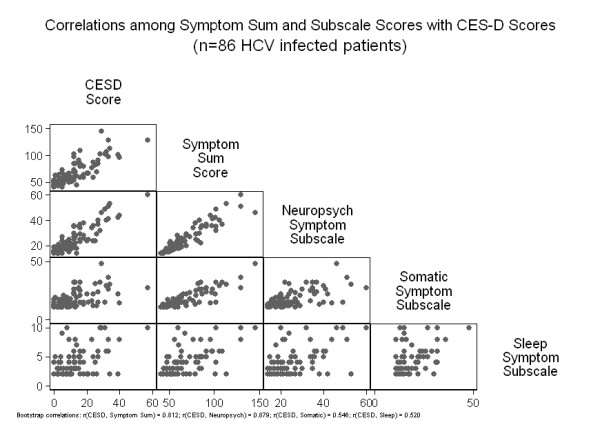
**Correlation matrix displaying the correlations among symptom sum and subscale scores with CES-D scores at baseline of HIV-infected individuals referred for assessment of hepatitis C treatment eligibility**.

Factor analysis of the 41-items HCV symptom inventory yielded a three-factor structure with eigenvalues ≥ 3.2, explaining 60% of the total variance. The factor accounting for the highest proportion of the variance (28%) included 14 items with strong loadings that corresponded to questions related to sadness, mood swings, anxiety, irritability, anhedonia, lack of concentration, and strange thoughts and was therefore termed the 'neuropsychiatric symptoms' factor. The second included 11 items that explained 22% of the variance and corresponded to questions related to body aches, dizziness, skin/hair changes, and nausea and was therefore named 'somatic symptoms' factor. The third, with high loadings for insomnia and interrupted sleep related questions explained 11% of the variance and was termed the 'sleep symptoms' factor. The final determination regarding scale inclusion of items was based on factor loadings and investigator judgment regarding content and face validity for a putative subscale construct (Table 1). Internal consistency reliability estimates of the 3 subscales were 0.93, 0.89 and 0.79 for neuropsychiatric, somatic and sleep symptoms, respectively. To examine convergent validity of the HCV symptom inventory subscales we compared the correlation between the HCV symptom inventory scores and CES-D scores. There was a strong correlation between the total symptom score and CES-D before HCV treatment initiation, with symptoms reported in the inventory accounting for 64% of the variability in the CES-D scores, r = 0.80 with 95% confidence interval(CI) 0.70 to 0.85, p < 0.00001 (Figure 1). Neuropsychiatric, somatic and sleep subscale score correlations (r) with CES-D scores were 0.88 (95% CI: 0.81 to 0.91), 0.51 (95% CI: 0.34 to 0.64) and 0.50 (95% CI: 0.32 to 0.63), respectively. Bootstrap comparison showed that the correlation of neuropsychiatric symptom score with CES-D scores was significantly different from the correlations of somatic and sleep symptom scores with CES-D scores (p = 0.001). However the magnitude of somatic and sleep symptoms correlations with CES-D did not differ significantly from one another (p = 0.89).

**Table 1:** Factor loadings in each of the items of the symptom inventory on the three factors extracted, after varimax rotation

Symptoms	Neuropsychiatric symptoms	Somatic symptoms	Sleep symptoms
Anxiety	0.52^a^	0.18	0.32
Cough or nasal congestion	0.24	0.24	0.01
Sadness/depression	0.71^a^	0.06	0.30
Restlessness	0.52^a^	0.19	0.42
No interest in activities	0.72^a^	0.05	0.32
Difficulty making decisions	0.74^a^	0.14	0.23
Strange thoughts	0.62^a^	-0.14	0.37
All-over sick feeling	0.42	0.63^a^	0.19
Difficulty getting to sleep	0.22	0.29	0.68^a^
Difficulty staying asleep	0.25	0.28	0.70^a^
Sleeping too much	0.45	0.10	-0.25
Nausea	0.38	0.55^a^	0.12
Vomiting	0.10	0.38	0.26
Loss of appetite	0.41^a^	0.15	0.10
Tiredness/fatigue	0.55^a^	0.38	0.15
Distractibility	0.75^a^	0.33	0.04
Body aches	0.26	0.72^a^	0.26
Joint pain	0.24	0.63^a^	0.30
Chest pain	0.14	0.64^a^	0.12
Other pain	0.09	0.52^a^	0.27
Episodes of confusion	0.70^a^	0.31	0.07
Word finding problem	0.59^a^	0.33	-0.04
Memory problem	0.66^a^	0.36	0.05
Irritability	0.57^a^	0.21	0.44
Decreased motivation	0.73^a^	0.21	0.13
Hallucinations	0.34	-0.01	0.52^a^
Lack of emotions	0.47	0.12	0.10
Mood swings	0.57^a^	0.32	0.47
Slowed movements	0.30	0.65^a^	-0.03
Tremor/shakiness	0.16	0.38	0.28
Walking problems	0.14	0.65^a^	0.33
Vision problems	0.02	0.47	0.04
Bladder problems	0.29	0.36	0.20
Loss of interest in sex	0.47	0.21	0.07
Fever	0.04	0.37	0.18
Headaches	0.50^a^	0.39	0.21
Nightmares/dreams	0.38	0.43	0.25
Shortness of breath	0.59^a^	0.47	-0.13
Rash/skin change/itching	0.19	0.68^a^	-0.02
Change in hair	-0.001	0.51^a^	0.29
Dizziness	0.32	0.55^a^	0.13

a = Item included in final factor structure.

### HCV symptom predictive validity

**Figure 2:**
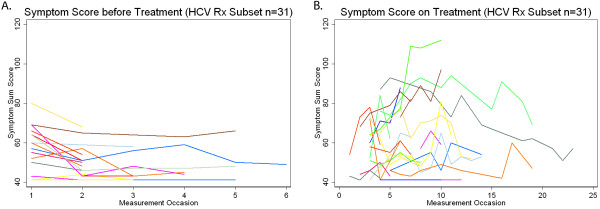
**Illustration of Individuals total sum symptom scores of HIV-infected patients before Hepatitis C treatment initiation (Panel A) and while on hepatitis C therapy (panel B)**.

Examination of the predictive validity of the HCV symptom inventory was conducted using generalized estimating equations modeling of the data scores for the 32 HCV/HIV patients who initiated HCV therapy. The median number of times that the symptom inventory was completed by each patient while on HCV therapy was 6 (range: 1-14). As shown in Table 2, patients developed worsening somatic and neuropsychiatric symptoms after HCV therapy initiation but not worsening sleep symptoms or CES-D scores. When the HCV symptom inventory sum scores were observed individually, they also worsened after HCV therapy initiation as shown in Figure 2. The coefficients (Bo, B1) of the models presented in Table 2 may be interpreted using the following example for HCV symptom inventory sum score as the dependent variable. For patients not on HCV therapy (reference group), the average total symptom score was 53.27. For patients on HCV therapy the average total symptom score was 60.19 (53.27 + 6.92).

**Table 2:** Unadjusted Generalized estimating equations models of treatment period (pre therapy vs. on HCV therapy)

Model	Score	**B**_**0**_ (Constant)	**B**_**1**_ (coefficient)	95% confidence interval	P
1	Sum symptoms	53.27	6.92	2.23 to 11.61	0.004
2	CES-D	7.31	1.86	-0.01 to 9.36	0.05
3	Neuropsychiatric symptoms	18.26	2.55	0.73 to 4.38	0.006
4	Somatic symptoms	14.30	2.57	1.01 to 4.14	0.001
5	Sleep symptoms	2.93	0.45	-0.08 to 0.98	0.09

Effects on symptoms and CES-D scales cores (n = 31).

CES-D = Center for Epidemiologic Studies Depression Scale

Each model had the form:

score = B_0 _+ B_1 _(treatment period); where treatment = 1 if on therapy and 0 if before therapy.

### Predictors of HCV therapy

During the study period 32 HIV-infected patients initiated therapy for HCV. Of those treated, 63% (n = 20) had liver biopsies. Using the Modified Knodell Score, liver biopsy showed absent/mild (F0-F2), moderate (F3-F4), and advanced fibrosis (F5) in 9, 8 and 3 patients respectively. Comparing those treated to the untreated, the median [range] ALT values were 52 [22-196] and 57 [23-302], respectively (p = 0.76). In bivariate analyses, there were no differences in HCV genotype or viral load, HIV risk factor, CD4 cell count, race/ethnicity, proportion of patients with psychiatric histories, or prior or current illicit substance use comparing HCV treated to not treated patients (Table 3). However, when race/ethnicity was categorized as a dichotomous variable (white vs. non-white), whites were more likely to initiate HCV therapy (p = 0.01), (Table 3). Of note, patients with a history of neuropsychiatric disease who were considered not eligible for HCV therapy had higher symptom sum scores than their counterparts with prior psychiatric histories who initiated HCV treatment (78.7 vs. 59.6, p = 0.009). In unadjusted analysis, predictors of HCV therapy initiation were lower HIV log_10 _RNA (1.66 vs. 2.12, p = 0.01), lower total symptom score (56.2 vs. 72.5, p = 0.0001), neuropsychiatric score (19.3 vs. 27.6, p = 0.00006), somatic score (18.9 vs. 16.1, p = 0.0001) sleep score (3.2 vs. 4.3, p = 0.04), and lower CES-D score (7.7 vs. 17.7, p = 0.0003) (Table 3). Backward stepwise multiple logistic regression analysis was used to identify independent predictors of treatment initiation in two models. The first model included only the HCV symptom inventory sum and subscale scores. The second model included all variables found to be significant (p < 0.10) in bivariate analysis (including race/ethnicity as dichotomous variable). In both stepwise logistic models, the most important predictor of HCV treatment initiation was neuropsychiatric symptom score, with an odds ratio (OR) of 0.88 (95% CI: 0.81 to 0.96, p = 0.002) in the first model, and OR: 0.86 (95% CI 0.79 to 0.94, p = 0.001) in the second model for each unit of increment in neuropsychiatric symptom score. HIV log_10 _RNA was also retained in the second model and was inversely associated with HCV treatment initiation with OR: 0.03 (95%CI: 0.0004 to 2.24, p = 0.11) for each increment of 1 log_10 _of HIV RNA.

**Table 3:** Bivariate analyses displaying predictors of Hepatitis C therapy in HIV co-infected patients

Variable	HCV treatment subset		P Value
	**No (n = 94)**	**Yes (n = 32)**	

Gender:			
Female	14	7	0.56
Male	79	25	
Race/ethnicity:			
White	55	11	0.09
Hispanic	20	9	
Black	17	10	
Unknown/other	2	2	
Race/ethnicity, dichotomized:			
White	55	11	0.02
Non-white	39	21	
HIV risk factor:			
Gay/bisexual	34	13	0.96
Heterosexual	11	3	
Intravenous drug use	39	13	
Hemophiliac	1	0	
Unknown/other	0	3	
CD4 cell count (×10^6^/l), mean (sd)	488(294)	478(272)	0.61
HIV log_10 _viral load, mean(sd)	2.12(0.96)	1.66(0.07)	0.01
HCV log_10 _RNA, mean (sd)	6.36(1.30)	6.27(1.12)	0.63
HCV genotype:			
Genotype 2 & 3	14	6	0.65
Genotype 1 & 4	74	25	
History of neuropsychiatric disease (%)	56 (60)	18 (56)	0.74
Ever use illicit substance (%)	87 (93)	28 (88)	0.38
Ongoing illicit substance use (%)	12 (13)	3 (9)	0.61
Total symptoms score, mean (sd)	73(25)	56(10)	0.002
Neuropsychiatric symptoms score, mean(sd)	28(11)	19(4)	0.00006
Somatic symptoms score, mean (sd)	19(8)	16(5)	0.0001
Sleep symptoms score, mean (sd)	4(3)	3(2)	0.04
CES-D score, mean (sd)	17(13)	8(7)	0.00003

CES-D = Center for Epidemiologic Studies Depression Scale.

## Discussion

The present study examined a quantitative assessment of symptoms that were hypothesized to be associated with the decision to initiate or defer HCV treatment initiation, independent of standard of care clinical considerations. The assessment was part of a multidisciplinary approach implemented within a comprehensive HIV primary care clinic caring for more than 3,000 patients of which 27% are co-infected with HCV.

We used a self-administered computer assisted symptom questionnaire completed just prior to the patient-provider encounter. Patient reported symptoms are important components of comprehensive clinical assessment [22]. Recently, a study of cancer patients found that internet assessment of different symptoms supplement traditional office visit discussions and fill important gaps in clinicians' knowledge, significantly improving patient safety and quality of care [23]. This suggests improvement in sensitivity of ascertainment when traditional methods are replaced by technologically enhanced symptoms assessment [24].

The study results suggest that the HCV symptom inventory could be a useful tool for consideration of HCV treatment eligibility in HIV infected patients. The HCV symptom inventory has a meaningful internal structure with excellent reliability as well as initial evidence supporting construct, convergent and predictive validity. The underlying structure of the inventory was factor analyzed into 3 primary constructs reflecting neuropsychiatric, somatic and sleep symptoms.

Similar to other reports [5,6,25,26], the present study found that severe neuropsychiatric symptoms and high CES-D scores at baseline were associated with independent judgment regarding treatment candidacy, perhaps due to clinician awareness of the likely worsening effect that interferon may have on neuropsychiatric symptoms [27,28]. A novel observation was that patients with worse somatic and sleep symptoms at baseline were less likely to be selected for HCV therapy initiation. Our results highlight the importance of baseline screening for somatic and sleep symptoms, particularly those that are known to be aggravated by HCV therapy: body aches, different somatic non-specific pains, skin complaints and nausea. Our results are in agreement with prior results validating the CES-D scale for HCV therapy and also identifying a somatic component in its underlying structure [21]. However, the CES-D somatic component has only 3 items (poor appetite, keep in mind what I was doing, and everything an effort to do) and does not focus specifically on comprehensive symptom evaluation.

The HCV symptom inventory could be viewed as a screening tool complementary to a well validated depression scale such as CES-D during HCV treatment eligibility assessment. Recognizing, acknowledging and medically assessing the presence of somatic and sleep symptoms in addition to neuropsychiatric screening prior to HCV therapy in HIV patients may enhance patient engagement in care and compliance with HCV therapy.

We found no effect of HCV genotype, viral load (both of which are predictors of HCV treatment response), CD4 cell count, HIV risk factor and gender on HCV treatment candidacy in the present study. This is consistent with the inclusive approach of the study team to treat as many HCV/HIV co-infected patients as possible. Our program aim is to also treat homeless individuals, patients with past and current substance use, and those with ongoing psychiatric conditions as long as they remain engaged in care. Consistent with prior reports [5,7], in our cohort whites tend to be treated more frequently that non-whites in unadjusted analysis. However, after adjusting for HIV viral load and neuropsychiatric symptom score, race was no longer associated with a treatment initiation decision. This likely reflected the clinician's perception of the decreased likelihood of HCV treatment response in non-white individuals when other known factors are taken in consideration [29]. The study was conducted before the interleukin-28B gene polymorphism was widely available as a screening tool to further characterize likelihood of HCV treatment response among non-white patients [30,31]. We noted that patients with higher HIV viral loads were less likely to be treated, but these patients had recently re-initiated HIV therapy. Interestingly, we did not find a significant negative impact following HCV therapy initiation in sleep symptoms or CES-D scores; these treatment-emergent symptoms were routinely treated by either the treatment team or the primary care providers, thus attenuating the expected effects on self reported depressive and sleep symptoms.

Inference from the study is subject to several limitations. First, since only 74% of patients completed the symptom inventory at each HCV staging visit, bias by unmeasured patients with worsening symptoms cannot be excluded. However, there was no difference in the mean symptom scores between patients who completed the symptom inventory at every clinic visit and those who completed the inventory occasionally (48.3 vs. 45.1, p = 0.79). We believe the missing inventories were more prevalent at the beginning of the implementation of our new clinic model when patients were not familiar with the process. We have subsequently implemented a procedure whereby the medical assistant immediately notifies our substance abuse counselor when a patient is placed in the exam room to avoid delays in the completion of the symptom inventory. Second, we recognize that both measured and unmeasured co-morbidities may increase variability in symptom scores. However, our immediate study objective was to examine the extent to which systematically measured symptomatology predicts treatment initiation decisions, while our longer term objective is to assess the symptom inventory as a predictor of adherence and treatment completion. Third, although the sample size could be considered relatively small (n = 126) it was sufficient to demonstrate the reliability and preliminary validity of the symptom inventory. Fourth, although stepwise multiple regression methods are popular in medical research, we acknowledge that they may have potentially serious shortcomings for valid inference [32]. As we described above this is an exploratory analysis to validate the symptom inventory and not a randomized controlled clinical trial. Future longitudinal trials are needed to confirm of our model conclusions. Finally, because the symptom inventory was performed only in English, inference regarding the observed findings is limited only to our English-speaking population. However, we recently have implemented a Spanish version of the symptom inventory for our patients when needed.

In conclusion, a 41 item hepatitis-related symptom inventory was found to have a clinically meaningful 3-factor structure with excellent internal consistency reliability and predictive validity. Both symptom and CES-D scores were predictive of treatment initiation decisions. Future research will identify whether the symptom inventory is useful in predicting adherence and treatment completion.

## Competing interests

Dr Gish has consulting relationships with over 15 HCV related companies, involved in the treatment of HCV. Other authors have non- financial competing of interest.

## Authors' contributions

EC Clinical care with sample collection, study design, data analyses and manuscript preparation DW, MG, FT CB & BC, clinical care and sample collection. RG Implemented modified version of symptom inventory used in present study and critical feed-back in redaction of manuscript. Wm CM Clinical care with sample collection, study design, data analyses and manuscript preparation. All authors read and approved the final manuscript.
